# Change in triglyceride-glucose index predicts the risk of cardiovascular disease in the general population: a prospective cohort study

**DOI:** 10.1186/s12933-021-01305-7

**Published:** 2021-05-26

**Authors:** Anxin Wang, Xue Tian, Yingting Zuo, Shuohua Chen, Xia Meng, Shouling Wu, Yongjun Wang

**Affiliations:** 1 China National Clinical Research Center for Neurological Diseases, Beijing Tiantan Hospital, Capital Medical University, No.119 South 4th Ring West Road, Fengtai District, Beijing, 100070 China; 2 Department of Neurology, Beijing Tiantan Hospital, Capital Medical University, Beijing, China; 3 Department of Epidemiology and Health Statistics, School of Public Health, Capital Medical University, Beijing, China; 4 Beijing Municipal Key Laboratory of Clinical Epidemiology, Beijing, China; 5 Department of Cardiology, Kailuan Hospital, North China University of Science and Technology, 57 Xinhua East Road, Tangshan, 063000 China

**Keywords:** Triglyceride-glucose index, Longitudinal changes, Cardiovascular disease, Stroke, Myocardial infarction, Predictive value

## Abstract

**Background:**

Previous studies has shown a significant relationship between baseline triglyceride-glucose (TyG) index and subsequent cardiovascular disease (CVD). However, the effect of longitudinal changes in TyG index on the risk of CVD remains uncertain. This study aimed to investigate the association between change in TyG index and the risk of CVD in the general population.

**Methods:**

The current study included 62,443 Chinese population who were free of CVD. The TyG index was calculated as ln [fasting triglyceride (mg/dL) × fasting glucose (mg/dL)/2], and change in TyG index was defined as the difference between the TyG index in 2010 and that in 2006. Multivariable-adjusted Cox proportional hazard models and restricted cubic spline analysis were used to examine the association between change in TyG index and the risk of CVD.

**Results:**

During a median follow-up of 7.01 years, 2530 (4.05%) incident CVD occurred, including 2018 (3.23%) incident stroke and 545 (0.87%) incident myocardial infarction (MI). The risk of developing CVD increased with the quartile of change in TyG index, after adjustment for multiple potential confounders, the hazard ratios for the Q4 group *versus* the Q1 group were 1.37 (95% confidence interval [CI], 1.21–1.54) for the overall CVD, 1.38 (95% CI, 1.19–1.60) for stroke, and 1.36 (95% CI, 1.05–1.76) for MI. Restricted cubic spline analysis also showed a cumulative increase in the risk of CVD with increases in the magnitude of change in TyG index. The addition of change in TyG index to a baseline risk model for CVD improved the C-statistics (*P* = 0.0097), integrated discrimination improvement value (*P* < 0.0001), and category-free net reclassification improvement value (*P* < 0.0001). Similar results were observed for stroke and MI.

**Conclusions:**

Substantial changes in TyG index independently predict the risk of CVD in the general population. Monitoring long-term changes in TyG may assist with in the early identification of individuals at high risk of CVD.

**Supplementary Information:**

The online version contains supplementary material available at 10.1186/s12933-021-01305-7.

## Background

Insulin resistance (IR), which is a critical mechanism of the pathogenesis of diabetes mellitus, has been extensively demonstrated to be closely related to be the development of cardiovascular disease (CVD) [1–3]. IR has been reported to be associated with not only CVD risk factors, such as diabetes mellitus [4], hypertension [5], dyslipidemia [6], and obesity [7], but also an independent risk factor for CVD [1–3]. Therefore, the early detection and control of IR may contribute to the prevention of CVD.

The gold-standard method for the assessment of IR is the hyperinsulinemic-euglycemic clamp, but this technique is not commonly used in the clinical settings or in large population-based studies due to the complex testing process and expensive cost [8]. The homeostasis model assessment of IR (HOMA-IR), which is calculated using fasting insulin and glucose concentrations, is commonly used for testing IR. However, the circulating insulin concentration is not routinely measured in primary care settings, which also renders HOMA-IR is also inappropriate for large-scale studies [9]. Instead, the triglyceride-glucose (TyG) index, which is the product of triglyceride (TG) and fasting blood glucose (FBG), has appeared as a simple, cost-effective, reproducible, and reliable surrogate for insulin resistance. This index has been shown to highly correlate with the hyperinsulinemic-euglycemic clamp and HOMA-IR [10–12]. Furthermore, previous cohort studies have shown that high TyG index was a risk factor for incident CVD [13–17]. However, an inherent limitation of these previous studies is that the TyG index was evaluated at a single time point, no analysis has been carried out on how the TyG index varies in individuals over time or the longitudinal effects of such changes. Such an assessment might be more useful prognostically than the measurement TyG index at a single time point.

Therefore, in the present study, we aimed to characterize the association between change in TyG index and the subsequent risk of CVD and its subtypes in a large community-based prospective cohort study.

## Methods

### Study population

The Kailuan study is a prospective cohort study in the Kailuan community in Tangshan, China. The detailed study design and procedures have been described previously [18–20]. During June 2006 to October 2007, a total of 101,510 participants (81,110 men and 20,400 women; aged 18–98 years) were enrolled in the first survey (baseline) and underwent a comprehensive biennial health examination. All participants were then followed until their death or December 31, 2017. Change in TyG index was developed from 2006 to 2010 to predict CVD risk from 2010 to 2017 (Additional file 1: Figure S1). We excluded 3669 and 2042 participants who experienced myocardial infarction (MI) or stroke, respectively, in or prior to 2010, 30,971 participants who did not complete the survey at 2010, and 1282 and 1103 participants with missing data on FBG or TG at baseline or the survey at 2010. Ultimately, a total of 62,443 participants were enrolled in the present study (Additional file 1: Figure S2). The study was performed according to the guidelines of the Helsinki Declaration and was approved by the Ethics Committee of Kailuan General Hospital (approval number: 2006-05) and Beijing Tiantan Hospital (approval number: 2010-014-01). All the participants agreed to take part in the study and provided written informed consent.

### Data collection and definitions

Information regarding the demographic characteristics, lifestyle factors (smoking habits, alcohol intake, and physical activity), and medical history were collected via standardized questionnaire by trained staffs. Educational level was classified as illiterate or primary school, middle school, and high school or above. Income was categorized into > 800 and ≤ 800 yuan/month. Smoking and alcohol intake habits were stratified into never, former or current. Physical activity was classified as ≥ 4 times per week and ≥ 20 min at a time, < 80 min per week, or none. Body mass index (BMI) was calculated by dividing body weight (kg) by the square of height (m^2^). Blood pressure was measured in the in the seated position using a mercury sphygmomanometer, and the mean results of three measurements of the systolic blood pressure (SBP) and diastolic blood pressure (DBP) were recorded. All the blood samples were analyzed using an auto-analyzer (Hitachi 747, Hitachi, Tokyo, Japan) on the day of the blood draw. The biochemical indicators tested included FBG, serum lipids, serum creatinine, and high-sensitivity C-reactive protein (hs-CRP).

Hypertension was defined as SBP ≥ 140 mmHg or DBP ≥ 90 mmHg, any use of the antihypertensive drug, or a self-reported history of hypertension. Diabetes was defined as FBG ≥ 7.0 mmol/L, any use of glucose-lowering drugs, or a self-reported history of diabetes. Dyslipidemia was defined as any self-reported history or use of lipid-lowering drugs, or total cholesterol (TC) ≥ 5.17 mmol/L.

### Calculation of changes in TyG index

The TyG index was calculated as ln (fasting TG [mg/dL] × FBG [mg/dL]/2), as previously described [21, 22]. Change in TyG index was calculated as the TyG index at in 2010 minus that at baseline (2006).

### Assessment of outcomes

The primary outcome in the present study was the first occurrence of CVD events (stroke and MI). We defined CVD events as described previously [18, 23, 24]. The database of CVD diagnoses was obtained from the Municipal Social Insurance Institution and Hospital Discharge Register and was updated annually during the follow-up period. An expert panel collected and reviewed the annual discharge records from 11 hospitals in Kailuan community to identify patients who were suspected of CVD. Incident stroke was diagnosed based on neurological signs, clinical symptoms, and neuroimaging tests, including computed tomography or magnetic resonance, according to the World Health Organization criteria [25]. MI was diagnosed according to the criteria of the World Health Organization on the based on the clinical symptoms, changes in the serum concentrations of cardiac enzymes and biomarkers, and electrocardiographic results [18, 26].

### Statistical analysis

The participants were divided into four categories according to the quartile of change in TyG index. The baseline characteristics are presented as mean ± standard deviation (SD) or frequency with percentage as appropriate. Differences in the characteristics across changes in TyG index categories were tested using analysis of variance or the Kruskal–Wallis test for continuous variables according to distribution, and using Chi-square for categorical variables. The person-years were determined from the date when the message was collected at baseline to either the date of CVD, death, or the date of participating in the last examination in this analysis, whichever came first. The Kaplan–Meier method was performed to evaluate the incidence rate of CVD and its subtypes, and the differences among groups were evaluated using the log-rank test.

Cox proportional hazard regression model was applied to calculate the hazard ratios (HRs) and 95% confidence intervals (CIs) for CVD and its subtypes. The proportional hazard assumption was evaluated by visualization of Schoenfeld residuals and no potential violation was observed. Two models were constructed: model 1 was adjusted for age, sex, and TyG index at baseline; model 2 was additionally adjusted for educational level, income, smoking status, drinking status, physical activity, BMI, SBP, DBP, history of hypertension, diabetes mellitus, and dyslipidemia, use of antidiabetic drugs, lipid-lowering drugs, and antihypertensive drugs, high-density lipoprotein cholesterol (HDL-C), low-density lipoprotein cholesterol (LDL-C), and hs-CRP at baseline. *P*-values for trend were computed using the quartile of change in TyG index as the ordinal variable. To capture the dose–response relationship between change in TyG index and the risk of CVD, restricted cubic splines analysis was used, with four knots at the 5th, 35th, 65th, and 95th percentiles of change in TyG index distribution. The reference point for TyG index change was the median value (− 0.60) of the reference (Q1) group, the HR was adjusted for variable in Model 2 [27].

Additional analyses were performed to validate the robustness of the results. First, the competing risk model was applied to assess the associations between change in TyG index and the outcomes, with non-CVD death being regarding as a competing risk event. Second, restricted analysis was performed by excluding participants with an abnormal FBG (≥ 7.0 mmol/L) or TG (≥ 1.7 mmol/L) concentration at baseline.[21] Third, to explore the potential impact of reverse causality, we repeated the primary analysis using a 2-year lag period by excluding participants who developed CVD cases within the first 2 years of follow up. Subgroup analyses were conducted on the participants after stratification by age (< 60 or ≥ 60 years), sex, BMI (< 25 or ≥ 25 kg/m^2^), and FBG (< 5.6, 5.6–6.9, and ≥ 7.0 mmol/L) to identify any modification by these variables, interactions between subgroups were tested for using likelihood ratio tests, in which models with and without multiplicative interaction terms were compared. Additionally, we used the C statistics, integrated discrimination improvement (IDI), and net reclassification index (NRI) to evaluate the incremental predictive value of change in TyG index beyond conventional risk factors.

All analyses were performed using SAS version 9.4 (SAS Institute, Cary, NC, USA) and R software version 3.6.1 (R Core Team, Vienna, Austria). All the statistical tests were 2-sided, and *P* < 0.05 was considered statistical significance.

## Results

### Baseline characteristics of the participants

A total of 62,443 eligible participants were included, their mean age was 49.07 ± 11.84 years, and 76.59% were men. A comparison of the baseline characteristics between and the participants and non-participants due to missing the 2010 survey or incomplete data is presented in Additional file 1: Table S1. Significant differences were observed between the participants and non-participants with respect to age, sex, educational level, income, smoking, drinking, medical history, and laboratory indeices.

The baseline characteristics of participants according to the quartile of change in TyG index are presented in Table 1. Compared with participants in the Q1 group, participants in the other groups were more likely to be older, men, less well educated, to have a lower income, more current smokers and drinkers, a higher prevalence of hypertension, diabetes, and dyslipidemia, more likely to table antihypertensive agents and antidiabetic agents, had a high BMI, SBP, DBP, TC, LDL-C, and hs-CRP level, and a lower HDL-C level.

**Table 1:** Baseline characteristics of participants according to quartiles of changes in TyG index from 2006 to 2010

Characteristics	Overall	Quartiles of changes in TyG index				*P* value
		Q1 (< − 0.31)	Q2 (− 0.31 to 0.05)	Q3 (0.05–0.41)	Q4 (≥ 0.41)	
No. of participants	62,443	15,610	15,611	15,611	15,611	
Age, years	49.07 ± 11.84	47.35 ± 11.77	48.82 ± 11.98	49.96 ± 11.95	50.15 ± 11.46	< 0.0001
Men, n (%)	47,827 (76.59)	12,059 (77.25)	11,562 (74.06)	11,806 (75.63)	12,400 (79.44)	< 0.0001
High school or above, n (%)	13,614 (22.56)	3755 (25.09)	3649 (24.17)	3322 (21.98)	2888 (19.02)	< 0.0001
Income > 800 RMB/month, n (%)	8878 (14.72)	2385 (15.95)	2410 (15.98)	2090 (13.84)	1993 (13.14)	< 0.0001
Body mass index, kg/m^2^	25.03 ± 3.46	24.84 ± 3.44	24.86 ± 3.47	25.01 ± 3.46	25.40 ± 3.46	< 0.0001
Systolic blood pressure, mmHg	128.36 ± 19.81	126.14 ± 19.20	126.98 ± 19.40	128.71 ± 19.95	131.60 ± 20.24	< 0.0001
Diastolic blood pressure, mmHg	82.63 ± 11.41	81.42 ± 11.23	81.86 ± 11.16	82.78 ± 11.35	84.44 ± 11.65	< 0.0001
Current smoker, n (%)	20,552 (33.81)	5082 (33.28)	4739 (31.10)	4975 (32.67)	5756 (38.25)	< 0.0001
Current alcohol use, n (%)	23,413 (38.51)	5665 (37.06)	5429 (35.62)	5816 (38.19)	6503 (43.22)	< 0.0001
Active physical activity, n (%)	55,080 (91.46)	13,387 (89.74)	13,850 (91.92)	13,922 (92.31)	13,921 (91.85)	< 0.0001
Hypertension, n (%)	6035 (9.66)	1416 (9.07)	1485 (9.51)	1470 (9.42)	1664 (10.66)	< 0.0001
Diabetes mellitus, n (%)	1489 (2.38)	339 (2.17)	288 (1.85)	337 (2.16)	525 (3.36)	< 0.0001
Dyslipidemia, n (%)	3183 (5.10)	737 (4.72)	833 (5.34)	770 (4.93)	843 (5.40)	0.0166
Antihypertensive agents, n (%)	5185 (8.30)	1209 (7.75)	1273 (8.16)	1282 (8.21)	1421 (9.10)	0.0002
Antidiabetic agents, n (%)	1144 (1.83)	269 (1.72)	222 (1.42)	252 (1.61)	401 (2.57)	< 0.0001
Lipid-lowering agents, n (%)	465 (0.74)	102 (0.65)	134 (0.86)	108 (0.69)	121 (0.77)	0.1526
Total cholesterol, mmol/L	4.91 ± 1.13	4.90 ± 1.04	4.94 ± 1.02	4.97 ± 1.02	4.84 ± 1.40	< 0.0001
HDL cholesterol, mmol/L	1.56 ± 0.39	1.58 ± 0.40	1.56 ± 0.39	1.55 ± 0.39	1.53 ± 0.40	< 0.0001
LDL cholesterol, mmol/L	2.29 ± 0.89	2.25 ± 0.94	2.28 ± 0.86	2.31 ± 0.87	2.32 ± 0.89	< 0.0001
Hs-CRP, mg/dL	2.29 ± 6.37	2.14 ± 4.96	2.21 ± 5.37	2.23 ± 6.94	2.60 ± 7.79	< 0.0001

*LDL* low-density lipoprotein, *HDL* high-density lipoprotein, *hs-CRP* high-sensitivity C-reactive protein, *TyG* triglyceride glucose

### Association of change in TyG index with CVD and its subtypes

**Fig. 1:**
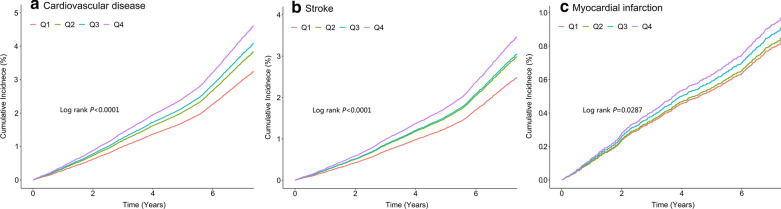
Kaplan–Meier estimation of (**A**) cardiovascular diseases (**B**) stroke (**C**) myocardial infarction by quartiles of changes in TyG index. *TyG* triglyceride-glucose

**Fig. 2:**
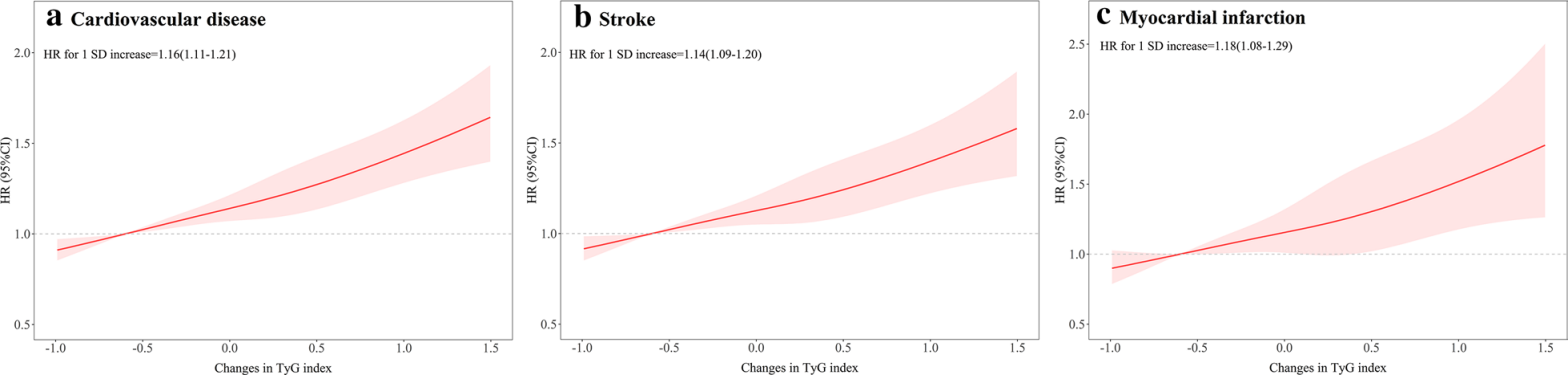
Multivariable-adjusted hazard ratios for (**A**) cardiovascular diseases (**B**) stroke (**C**) myocardial infarction based on restricted cubic spines with 5 knots at 5th, 25th, 50th, 75th, and 95th percentiles of changes in TyG index. *HR* hazard ratio, *SD* standard deviation, *TyG* triglyceride-glucose. Red line represent references for hazard ratios, and red area represent 95% confidence interval. Model was adjusted for age, sex, TyG index, education, income, smoking status, drinking status, physical activity, body mass index, systolic blood pressure, diastolic blood pressure, a history of hypertension, diabetes mellitus, and dyslipidemia, antidiabetic agents, lipid-lowering agents, antihypertensive agents, high-density lipoprotein cholesterol, high-density lipoprotein cholesterol, and high-sensitivity C-reactive protein at baseline

During a median follow-up of 7.01 years (interquartile range: 6.64–7.31 years), 2530 (4.05%) incident CVD were identified, including 2018 (3.23%) incident stroke and 545 (0.87%) incident MI. The incidence of CVD increased substantially with the magnitude of change in TyG index (quartiles), reaching a maximum incidence of 6.73 (95% CI, 6.25–7.24) per 1000 person-years in Q4. The cumulative risk of CVD also increased according to the magnitude of change in TyG index (Fig. 1A), and this trend remained significant even after adjustment for potential confounding factors in model 2 (*P* for trend < 0.001), the HRs were 1.18 (95% CI, 1.06–1.32), 1.26 (95% CI, 1.12–1.42), and 1.42 (95% CI, 1.26–1.60) for the Q2, Q3, and Q4 groups versus the Q1 group of change in TyG index (Table 2). Moreover, there was a linear relationship between change in TyG index and risk of CVD, per 1 SD increase in change in TyG was associated with a 16% higher risk of CVD (HR, 1.16; 95% CI, 1.11–1.21; Fig. 2A). In the subtype analyses for CVD, similar results were yield for stroke and MI, with the HRs increasing across increasing change in TyG quartiles (Table 2; Figs. 1B, C, 2B, C).

**Table 2:** HR and 95% CI for the association between changes in TyG index from 2006 to 2010 and cardiovascular diseases and its subtypes

	Quartiles of changes in TyG index				*P* for trend
	Q1 (< − 0.31)	Q2 (− 0.31 to 0.05)	Q3 (0.05–0.41)	Q4 (≥ 0.41)	
CVD					
Case, n (%)	585 (3.75)	594 (3.81)	646 (4.14)	705 (4.52)	
Incidence rate, per 1000 person-y	5.54 (5.11–6.01)	5.65 (5.21–6.12)	6.17 (5.71–6.66)	6.73 (6.25–7.24)	
Model 1	Reference	1.18 (1.06–1.32)	1.26 (1.12–1.42)	1.42 (1.26–1.60)	< 0.0001
Model 2	Reference	1.17 (1.04–1.30)	1.24 (1.11–1.40)	1.37 (1.21–1.54)	< 0.0001
Stroke					
Case, n (%)	465 (2.98)	473 (3.03)	528 (3.38)	552 (3.54)	
Incidence rate, per 1000 person-y	4.39 (4.01–4.80)	4.48 (4.10–4.91)	5.03 (4.62–5.47)	5.24 (4.82–5.70)	
Model 1	Reference	1.24 (1.08–1.41)	1.27 (1.11–1.47)	1.44 (1.24–1.66)	< 0.0001
Model 2	Reference	1.22 (1.07–1.40)	1.26 (1.09–1.45)	1.38 (1.19–1.60)	< 0.0001
MI					
Case, n (%)	126 (0.81)	126 (0.81)	128 (0.82)	165 (1.06)	
Incidence rate, per 1000 person-y	1.18 (0.99–1.40)	1.19 (1.00–1.41)	1.21 (1.02–1.44)	1.55 (1.33–1.81)	
Model 1	Reference	1.05 (0.82–1.34)	1.22 (0.95–1.57)	1.41 (1.09–1.82)	0.0050
Model 2	Reference	1.02 (0.80–1.30)	1.19 (0.93–1.53)	1.36 (1.05–1.76)	0.0115

Model 1: adjusted for age, sex, and TyG index at baseline. Model 2: further adjusted for education, income, smoking status, drinking status, physical activity, body mass index, systolic blood pressure, diastolic blood pressure, a history of hypertension, diabetes mellitus, and dyslipidemia, antidiabetic agents, lipid-lowering agents, antihypertensive agents, high-density lipoprotein cholesterol, high-density lipoprotein cholesterol, and high-sensitivity C-reactive protein at baseline

*CI* confidence interval, *CVD* cardiovascular disease, *MI* mypcardial infarction, *HR* hazard ratio, *TyG index* triglyceride-glucose index

**Fig. 3:**
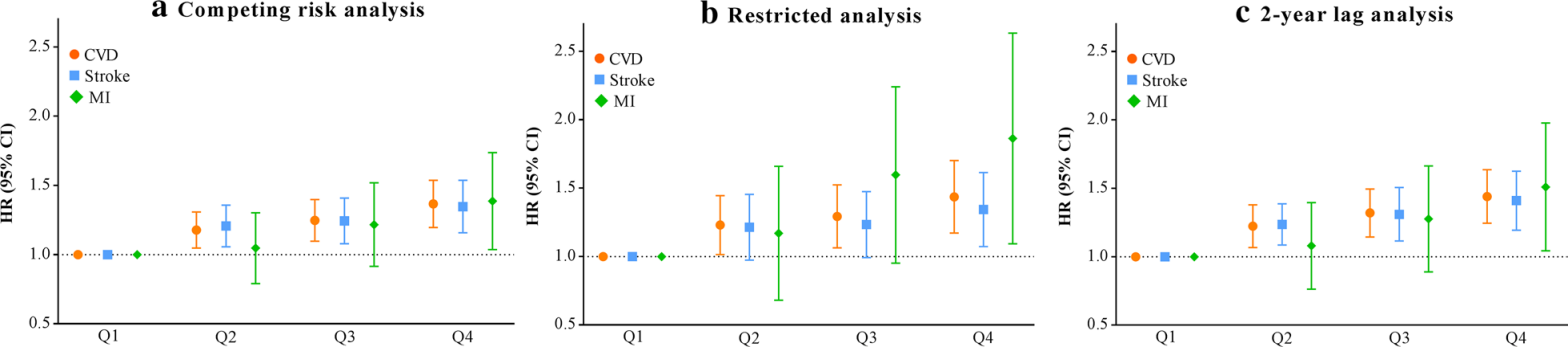
Sensitivity analyses for the association of changes in TyG index from 2006 to 2010 with cardiovascular disease and its subtypes. *CVD* cardiovascular disease, *MI* myocardial infarction, *TyG index* triglyceride-glucose index. Model was adjusted for age, sex, TyG index, education, income, smoking status, drinking status, physical activity, body mass index, systolic blood pressure, diastolic blood pressure, a history of hypertension, diabetes mellitus, and dyslipidemia, antidiabetic agents, lipid-lowering agents, antihypertensive agents, high-density lipoprotein cholesterol, high-density lipoprotein cholesterol, and high-sensitivity C-reactive protein at baseline. **A** Taking non-CVD related death as competing risk event rather than censoring. **B** Restricted analysis was excluded those with abnormal FBG (≥ 7.0 mmol/L) or abnormal TG level (≥ 1.7 mmol/L) at baseline (n = 21,901). C. Excluded person time and incident CVD cases from the first 2 years of follow-up (n = 1162)

The sensitivity analyses with competing risk model (Fig. 3A), excluding participants with abnormal FBG or TG level at baseline (n = 21,901, Fig. 3B), and by excluding the outcome events that occurred within the first 2 years of the follow-up period (n = 1162, Fig. 3C), all generated similar findings to the primary analysis.

### Subgroup analyses

The results of the subgroup analyses are presented in Additional file 1: Table S2. The association of change in TyG index with the risk of CVD and its subtypes were consistent across the subgroups, including age, sex, BMI, and FBG. There were no significant interactions between changes in TyG index and the stratified variables (*P* for interaction > 0.05 for all).

### Incremental predictive value of changes in TyG index

Finally, we evaluated whether changes in TyG index would further increase the predictive value of conventional risk (Table 3). The C statistics by the conventional model significantly improve with the addition of change in TyG index (from 0.739 to 0.742, *P* = 0.0097), the discriminatory power and risk reclassification also appeared to be substantially better, with the IDI of 0.09% (95% CI, 0.05–0.13; *P* < 0.0001), and the NRI of 12.58% (95% CI, 8.61–16.56; *P* < 0.0001). Similar results were observed when stroke and MI.

**Table 3:** Reclassification and discrimination statistics for changes in TyG index

	C statistics		IDI		Category-free NRI	
	Estimate (95% CI)	*P*	Estimate (95% CI), %	*P*	Estimate (95% CI), %	*P*
CVD						
Conventional model^a^	0.739 (0.731–0.748)		Reference		Reference	
Conventional model + changes in TyG index	0.742 (0.733–0.751)	0.0097	0.09 (0.05–0.13)	< 0.0001	12.58 (8.61–16.56)	< 0.0001
Stroke						
Conventional model^a^	0.740 (0.730–0.750)		Reference		Reference	
Conventional model + changes in TyG index	0.742 (0.732–0.752)	0.0435	0.06 (0.02–0.09)	0.0010	10.83 (6.40–15.26)	< 0.0001
MI						
Conventional model^*^	0.749 (0.731–0.766)		Reference		Reference	
Conventional model + changes in TyG index	0.752 (0.735–0.770)	0.0412	0.03 (0.01–0.05)	0.0365	16.69 (8.26–25.12)	0.0001

*CVD* cardiovascular disease, *IDI* integrated discrimination improvement, *MI* myocardial infarction, *NRI* net reclassification index, *TyG index* triglyceride-glucose index

^a^Conventional model was adjusted for age, sex, TyG index, education, income, smoking status, drinking status, physical activity, body mass index, systolic blood pressure, diastolic blood pressure, a history of hypertension, diabetes mellitus, and dyslipidemia, antidiabetic agents, lipid-lowering agents, antihypertensive agents, high-density lipoprotein cholesterol, high-density lipoprotein cholesterol, and high-sensitivity C-reactive protein at baseline

## Discussion

In this prospective cohort study, we found that change in TyG index was significantly associated with the risk of CVD. Notably, the risk of CVD increased with the increase in TyG index over time. Similar patterns were observed for stroke and MI. These trends remained when subjected to multiple sensitivity analyses and in analyses of stratified data. Furthermore, the addition of change in TyG index to the baseline risk model including traditional risk factors, comprising conventional risk factors, significantly promoted the ability of risk stratification.

The present analyses showed that participants with an increasing TyG index were at a higher risk of developing CVD relative to their counterparts with a decreasing TyG index over time. Previous studies, which generally used a single measurement of TyG index assessment generated consistent results regarding the association between a single TyG index value and subsequent CVD risk. The Vascular Metabolic CUN cohort, comprising 5,014 subjects, found that a higher level of TyG index was significantly associated with higher risk of developing CVD in a Caucasian population, with participants in the highest quintile group having a 2.32-fold higher risk of developing CVD than those in the lowest quintile group [13]. Another retrospective cohort study of 6078 participants who were aged over 60 years showed that participants in the highest quartile of TyG index were at a 72% higher risk of CVD events [14]. The Tehran Lipid and Glucose Study of 7521 Iranians revealed that the significant relationship between the TyG index and the risk of CVD/coronary heart disease was more prominent among the younger population [15]. Finally, data from the National Health Information Database showed that participants in the highest TyG index quartile were at higher risks of stroke and MI, independently of other traditional cardiovascular risk factors [16].

Of note, the TyG index is calculated using the TG and FBG concentrations, both of which vary over time. Therefore, the evaluation of the TyG index at baseline alone does not reflect the longitudinal association between the dynamic changes in TyG index and CVD risk. Moreover, a single measurement of the TyG index is also subject to potential regression dilution bias and reverse causation issue [28]. To address these knowledge gaps and methodological limitations, the concept of assessing the effect of change in TyG index on clinical outcomes has been proposed. In a cohort study conducted in rural China, the differences in TyG index between baseline and a subsequent examination was used to predict the risk of type 2 diabetes, and the results showed that the risk of incident diabetes was increased with the quartile of TyG difference in normal-weight people [29]. The TyG index was correlated to HOMA-IR and was reported to be better associated with atherosclerotic diseases than HOMA-IR. Therefore, we considered that the associations of CVD with HOMA-IR might also be reflected in a relationship with TyG index. The Tehran lipid and glucose study showed that changes in IR assessed using HOMA-IR were significantly associated with the development of hypertension by previously normotensive individuals [30]. Furthermore, the Slow the Adverse Effects of Vascular Aging trial showed that young overweight and obese adults who manage to reduce their insulin concentration, as well as their HOMA-IR over 6 months showed less vascular stiffness [31]. Nevertheless, the relationship between change in TyG index and CVD has not been investigated in previous studies. Consistent with above-mentioned study, our study found that the risk of CVD was increased with the quartile of change in TyG index, which suggests that participants who experience a large increase in TyG index may warrant particular vigilance and should be followed closely in case they develop CVD.

Another important finding of the present study was that the addition of change in TyG to the conventional risk model had an incremental effect on the predictive value for incident CVD. The predictive utility of a single TyG index value for the prediction of CVD has been showed in previous studies. Data from the Kaohsiung Medical University Hospital showed that the TyG index is a useful parameter and a stronger predictive factor of cardiovascular events than hemoglobin A1c, thus may be of additional prognostic benefit in patients with type 2 diabetes [17]. A study of the Vascular Metabolic CUN cohort showed that the areas under the curve of receiver -operating characteristics curve was increased from 0.708 to 0.719 by adding the TyG index to the Framingham model [13]. Finally, the present findings imply that a substantial increase in TyG index is associated with a high risk of CVD, and that this higher risk can be predicted more accurately than with the models that include a single TyG index value, which highlights the importance of monitoring longitudinal patterns of changes in TyG index in clinical practice.

The mechanism underlying the associations of change in TyG index with the development and progression of CVD remains uncertain, but several possibilities have been proposed. First, a previous study showed that FBG mainly reflects IR in the liver, whereas fasting TG mainly reflects IR in adipocytes [32]. Therefore, it can be postulated that an increase in TyG index over time may reflect IR affecting both of these organs. IR plays an important role in the formation of atherosclerotic plaques because it leads to chronic inflammation, oxidative stress, and endothelial dysfunction, facilitates the formation of foam cells, and changes the expression of the estrogen receptor, as shown in animal models [33–36]. Second, in the present study, participants who showed substantial changes in TyG index tended to also have more severe and complex disease, as defined using their BMI, blood pressure, lipid profile, hypertension, diabetes, and dyslipidemia, which are risk factors for CVD [37, 38]. A change in TyG index might modify the effects of other cardiovascular risk factors and contribute to the progression of CVD. Third, it has been demonstrated that the TyG index is related to arterial stiffness, using measurements of pulse wave velocity, ankle-brachial index, and carotid intima-media thickness, through effects on platelet adhesion, activation, and aggregation [39, 40]. Thus an increase in TyG index over time may accelerate the development of arterial stiffness, thereby leading to the development of CVD.

The strengths of the present study include its prospective design, the large community-based sample, long follow-up period, and the assessment of the effect of change in TyG index on incident CVD and its subtypes in the general population. The present study also had several limitations. First, owing to a shortage of records insulin concentration data, we could not compare the predictive value of TyG index with those of HOMA-IR and the hyperinsulinaemic euglycaemic clamp test for the development of CVD. Second, the sex distribution of the sample was unbalanced because a large proportion of the participants were coal miners. However, the association of change in TyG index with CVD and subtypes were statistically robust, given that a significant interaction was not identified when data were stratified according to sex. Third, owing to the observational nature of the study, we could not establish a causal link between TyG index and the risk of CVD, and therefore the present findings should to be confirmed in future studies. Finally, although other potential cardiac risk factors were adjusted for, we still cannot exclude the possibility of residual or unassessed confounding given the observational nature of the present analysis.

## Conclusions

We found that change in TyG index is an independent predictor of CVD and its subtypes. An increase in TyG index over time isassociated with higher risks of CVD, stroke and MI. These findings emphasize the importance of monitoring the longitudinal changes in TyG index to most effectively identify individuals who are at high risk of developing CVD.

## Supplementary Information

**Additional file 1**: **Table S1**. Comparison of baseline characteristics of participants and non-participants due to missing data. **Table S2**. Subgroup analyses for the association of changes in TyG index with cardiovascular disease and its subtypes. **Figure S1**. Timeline of the study. **Figure S2**. The flowchart of the study.

## Data Availability

The datasets used and/or analyzed during the current study are available from the corresponding author on reasonable request.

## Abbreviations

BMI: Body mass index
CI: Confidence interval
CVD: Cardiovascular disease
DBP: Diastolic blood pressure
FBG: Fasting blood glucose
HDL-C: High-density lipoprotein cholesterol
HOMA-IR: Homeostasis model assessment insulin resistance
HR: Hazard ratio
hs-CRP: High-sensitivity C-reactive protein
IDI: Integrated discrimination improvement
LDL-C: Low-density lipoprotein cholesterol
NRI: Net reclassification index
SBP: Systolic blood pressure
SD: Standard deviation
TG: Triglyceride
TyG: Triglyceride-glucose
