# Supplementary material for: Change in triglyceride-glucose index predicts the risk of cardiovascular disease in the general population: a prospective cohort study
Source: Cardiovasc Diabetol. 2021 May 26;20:113. doi: 10.1186/s12933-021-01305-7 (PMC8157734; doi:10.1186/s12933-021-01305-7)
Supplement: Supplementary file 1 — Additional file 1: Table S1. Comparison of baseline characteristics of participants and non-participants due to missing data. Table S2. Subgroup analyses for the association of changes in TyG index with cardiovascular disease and its subtypes. Figure S1. Timeline of the study. Figure S2. The flowchart of the study. [file 12933_2021_1305_MOESM1_ESM.docx]

**Additional file 1**

Table S1. Comparison of baseline characteristics of participants and non-participants due to missing data

| Characteristics | Non-participants | Participants | *P* value |
| --- | --- | --- | --- |
| No. of participants | 33356 | 62443 |  |
| TyG index | 8.66±0.70 | 8.64±0.69 | <0.0001 |
| Age, years | 55.58±12.84 | 49.07±11.84 | <0.0001 |
| Men, n (%) | 27821 (84.80) | 47827 (76.59) | <0.0001 |
| High school or above, n (%) | 5018 (16.14) | 13614 (22.56) | <0.0001 |
| Income >800 RMB/month, n (%) | 4118 (13.25) | 8878 (14.72) | <0.0001 |
| Body mass index, kg/m^2^ | 24.94±3.55 | 25.03±3.46 | 0.0003 |
| Systolic blood pressure, mm Hg | 134.2±22.04 | 128.36±19.81 | <0.0001 |
| Diastolic blood pressure, mm Hg | 84.48±12.22 | 82.63±11.41 | <0.0001 |
| Current smoker, n (%) | 10972 (34.95) | 20552 (33.81) | 0.0005 |
| Current alcohol use, n (%) | 11199 (35.66) | 23413 (38.51) | <0.0001 |
| Active physical activity, n (%) | 28168 (90.75) | 55080 (91.46) | 0.0004 |
| Hypertension, n (%) | 4278 (13.04) | 6035 (9.66) | <0.0001 |
| Diabetes Mellitus, n (%) | 1073 (3.27) | 1489 (2.38) | <0.0001 |
| Dyslipidemia, n (%) | 1629 (4.96) | 3183 (5.09) | 0.3755 |
| Antihypertensive agents, n (%) | 3686 (11.24) | 5185 (8.30) | <0.0001 |
| Antidiabetic agents, n (%) | 805 (2.45) | 1144 (1.83) | <0.0001 |
| Lipid-lowering agents, n (%) | 198 (0.60) | 465 (0.74) | 0.0128 |
| Fasting plasma glucose, mmol/L | 5.61±1.86 | 5.38±1.53 | <0.0001 |
| Total cholesterol, mmol/L | 5.00±1.18 | 4.91±1.13 | <0.0001 |
| Triglycerides, mmol/L | 1.65±1.36 | 1.68±1.38 | 0.0048 |
| HDL cholesterol, mmol/L | 1.53±0.42 | 1.56±0.39 | <0.0001 |
| LDL cholesterol, mmol/L | 2.45±0.93 | 2.29±0.89 | <0.0001 |
| Hs-CRP,mg/dL | 2.50±6.58 | 2.29±6.37 | <0.0001 |

Abbreviations: LDL, low-density lipoprotein; HDL, high-density lipoprotein; hs-CRP, high-sensitivity C-reactive protein; TyG, triglyceride glucose.

Table S2. Subgroup analyses for the association of changes in TyG index with cardiovascular disease and its subtypes

| Variables | CVD | *P*_interaction_ | Stroke | *P*_interaction_ | MI | *P*_interaction_ |
| --- | --- | --- | --- | --- | --- | --- |
| Age, years |  |  |  |  |  |  |
| <60 | 1.21(1.12-1.31) | 0.7978 | 1.21(1.11-1.32) | 0.5801 | 1.17(0.99-1.38) | 0.1901 |
| ≥60 | 1.23(1.08-1.41) |  | 1.15(1.00-1.34) |  | 1.44(1.09-1.90) |  |
| Sex |  |  |  |  |  |  |
| Women | 1.18(0.95-1.47) | 0.2025 | 1.21(0.96-1.54) | 0.4021 | 1.05(0.94-1.67) | 0.1049 |
| Men | 1.27(1.19-1.37) |  | 1.24(1.15-1.35) |  | 1.22(1.15-1.55) |  |
| BMI, kg/m^2^ |  |  |  |  |  |  |
| <25 | 1.37(1.23-1.51) | 0.9864 | 1.32(1.18-1.49) | 0.9277 | 1.46(1.16-1.80) | 0.9926 |
| ≥25 | 1.20(1.10-1.31) |  | 1.18(1.07-1.31) |  | 1.23(1.02-1.48) |  |
| FBG, mmol/L |  |  |  |  |  |  |
| <5.6 | 1.21(1.11-1.33) | 0.5020 | 1.15(1.04-1.28) | 0.5504 | 1.37(1.13-1.65) | 0.9268 |
| 5.6-7.0 | 1.18(1.02-1.36) |  | 1.16(0.99-1.36) |  | 1.23(0.91-1.66) |  |
| ≥7.0 | 1.32(0.96-1.82) |  | 1.30(0.93-1.81) |  | 1.05(0.38-2.89) |  |

Abbreviation: CVD, cardiovascular disease; MI, mypcardial infarction; TyG index, triglyceride-glucose index.

Model was adjusted for age, sex, TyG index, education, income, smoking status, drinking status, physical activity, body mass index, systolic blood pressure, diastolic blood pressure, a history of hypertension, diabetes mellitus, and dyslipidemia, antidiabetic agents, lipid-lowering agents, antihypertensive agents, HDL cholesterol, LDL cholesterol, and hs-CRP at baseline other than variables for stratification.

Year 2006

Year 2008

Year

2010

TyG index

assessment period

Follow-up for outcomes ascertainment

End of follow-up

(December 2017)

**Figure S1. Timeline of the study.**

Abbreviation: TyG index, triglyceride-glucose index.


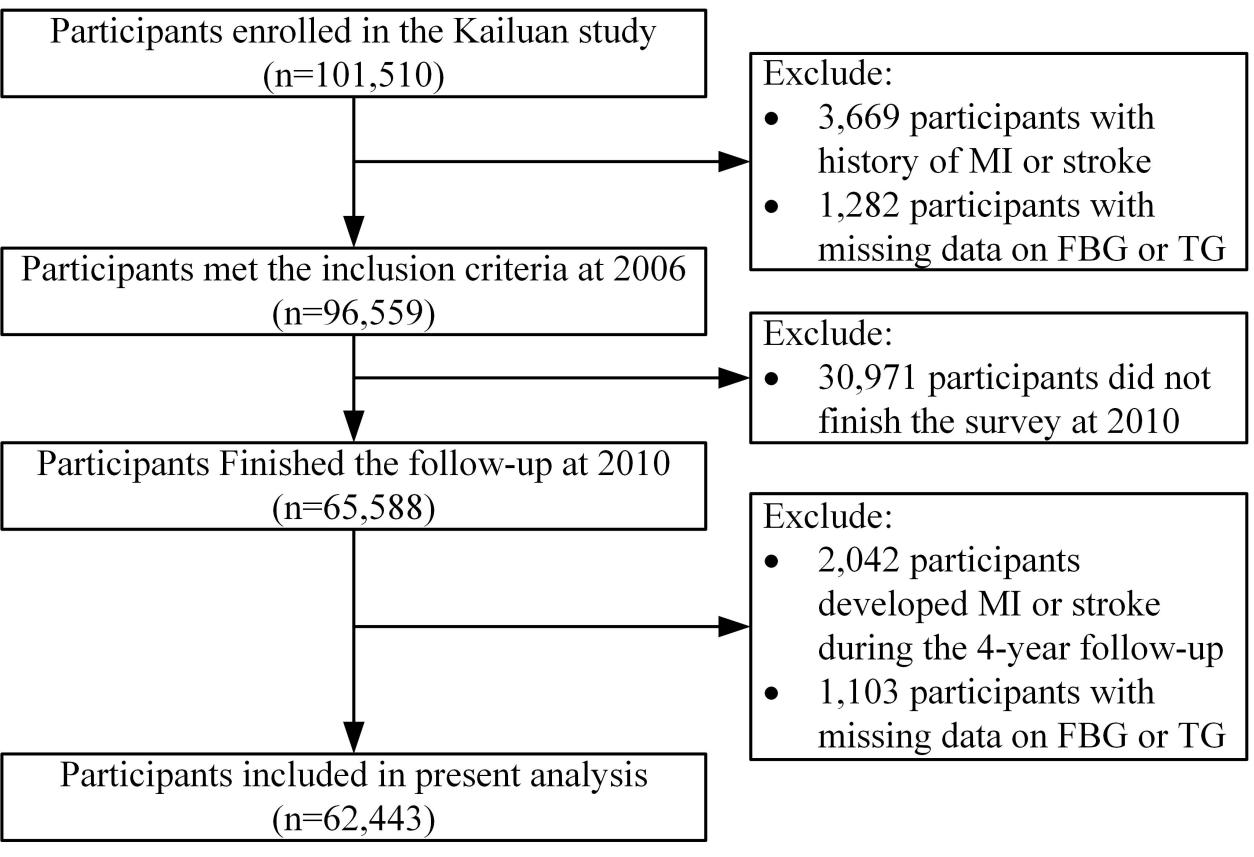


**Figure S2. The flowchart of the study**

Abbreviations: FBG, fasting blood glucose; MI, myocardial infarction; TG, triglyceride
